# The ability of surface topography postural measurements to detect cobb angle progression in adolescents with idiopathic scoliosis (AIS) and a main thoracic curve: full torso scans compared to back only parameters

**DOI:** 10.1186/1748-7161-9-S1-O10

**Published:** 2014-12-04

**Authors:** Eric C Parent, Samantha Chabot, Lindsey Westover, Douglas Hill, Marc Moreau, Douglas Hedden, Edmond Lou, Samer Adeeb

**Affiliations:** 1 University of Alberta, Edmonton, Canada; 2 Alberta Health Services and University of Alberta, Edmonton, Canada

## Background

External deformity due to scoliosis can be quantified by surface topography (ST) from full-torso and back-only scans. Determining the ability of ST parameters to detect which curves remain stable is necessary to determine if ST can help reduce radiation exposure in monitoring scoliosis progression.

## Objective

The study goal was to compare the ability of full-torso and back-only ST parameters to detect which curves do not progress by >5 (Cobb degrees) in AIS with a main thoracic curve.

## Design

Prospective cohort.

## Methods

We assessed 42 adolescents (n=32F, age 13.9±1.7yrs) with AIS with a main thoracic curve, braced (n=22) or under observation (n=20), using a full-torso ST scan at baseline and 12±3months later. Subjects were scanned standing in a positioning frame using four laser scanners. One evaluator marked 11 landmarks. Thirty full-torso and 16 back-only parameters were extracted in Matlab by digitizing landmarks on anonymized scans presented randomly. The absolute value of the difference between visits was quantified for ST changes because surface improvement and deterioration can occur with worsening curvatures. The area under the receiver operator characteristic curves(AUC) was used to compare the accuracy in determining which curves did not progress.2 An AUC of 1 represents a perfect and .5 a worthless parameter.

## Results

The baseline Cobb angle was 24±12 and the mean 1-yr change was 1.6±8.6 (range -10;34degrees). The largest curve worsened by >5 degrees for 13 patients. Two full-torso ST parameters had statistically significant ability to predict which curve remained stable. The AUC of the absolute of the change in “the 10th to 90th percentile range3 of the angle between the principal axis of inertia of torso cross-sections and the frontal plane” 1 was 0.70 (95%CI 0.52;0.88). The absolute change in the “transverse plane angle between the anterior superior iliac spines and the sternum” was 0.73 (95%CI 0.58;0.88). No back-only parameters demonstrated a significant ability to predict stable curves.

## Conclusion

In patients with main thoracic curves, only full-torso ST parameters had significant ability to detect stable curves during a 1 year follow-up. Future work will determine if a prediction rule using ST parameters can be developed to detect stable curves and reduce radiation exposure.
